# Supplementary material for: Effect of Parity, Body Condition Score at Calving, and Milk Yield on the Metabolic Profile of Gyr Cows in the Transition Period
Source: Animals (Basel). 2023 Aug 3;13(15):2509. doi: 10.3390/ani13152509 (PMC10417048; doi:10.3390/ani13152509)
Supplement: Supplementary file 1 [file animals-13-02509-s001.zip › SupplementaryTable S1 Breda et al. Metabolic profile of Gyr cows Animals abr 2023.pdf]

Supplementary Table S1. Variation (mean ± SD) of body condition score (BCS), serum concentrations of non-esterified fatty acids (NEFA), beta-hydroxybutyrate (BHB), and cholesterol, and plasma glucose concentrations in high-producing Gyr cows grouped according to parity, on different days relative to calving (0 d), throughout the transition period.

Variable	Group	-21 d	-7 d	0 d	7 d	21 d	42 d
BCS	P	$3.30±0.19^{Aa}$	$3.29±0.20^{Aa}$	$3.29±0.20^{Aab}$	$3.17±0.25^{Bb}$	$3.10±0.27^{Abc}$	$3.02±0.46^{Bc}$
	B	$3.38±0.15^{Aab}$	$3.42±0.14^{Aa}$	$3.42±0.14^{Aa}$	$3.35±0.17^{Aab}$	$3.25±0.16^{Ab}$	$3.20±0.19^{Ab}$
	M	$3.35±0.12^{Aa}$	$3.35±0.17^{Aa}$	$3.35±0.17^{Aa}$	$3.30±0.16^{ABa}$	$3.22±0.21^{Aa}$	$3.25±0.21^{Aa}$
NEFA (mmol/L)	P	$0.26±0.04^{Ab}$	$0.30±0.14^{Ab}$	$0.79±0.35^{Aa}$	$0.72±0.46^{Aab}$	$0.50±0.40^{Ab}$	$0.54±0.31^{Aab}$
	B	$0.23±0.13^{Ac}$	$0.29±0.22^{Ac}$	$0.80±0.33^{Aab}$	$0.96±0.66^{Aa}$	$0.55±0.39^{Abc}$	$0.63±0.51^{Ab}$
	M	$0.38±0.18^{Ab}$	$0.52±0.46^{Ab}$	$0.90±0.30^{Aa}$	$0.69±0.46^{Aab}$	$0.52±0.54^{Ab}$	$0.74±0.51^{Aab}$
BHB (mmol/L)	P	$0.47±0.10^{Ab}$	$0.48±0.11^{Ab}$	$0.43±0.19^{Ab}$	$0.68±0.24^{Aa}$	$0.57±0.29^{Aab}$	$0.44±0.12^{Bb}$
	B	$0.36±0.09^{Ab}$	$0.45±0.10^{Ab}$	$0.44±0.18^{Ab}$	$0.69±0.27^{Aa}$	$0.60±0.33^{Aab}$	$0.62±0.36^{Aab}$
	M	$0.36±0.13^{Ab}$	$0.43±0.15^{Ab}$	$0.48±0.10^{Ab}$	$0.64±0.23^{Aab}$	$0.46±0.22^{Ab}$	$0.69±0.56^{Aa}$
Glucose (mmol/L)	P	$2.14±1.15^{Ab}$	$3.06±0.63^{Ab}$	$6.64±2.17^{Aa}$	$3.43±0.74^{Ab}$	$3.55±0.60^{Ab}$	$3.47±0.67^{Ab}$
	B	$2.99±0.64^{Ab}$	$2.98±0.63^{Ab}$	$6.13±2.98^{Aa}$	$3.26±0.81^{Ab}$	$3.29±0.65^{Ab}$	$3.29±0.68^{Ab}$
	M	$3.05±0.45^{Ab}$	$2.98±0.37^{Ab}$	$6.12±3.04^{Aa}$	$3.52±0.75^{Ab}$	$3.58±0.92^{Ab}$	$3.10±0.92^{Ab}$
Cholesterol (mmol/L)	P	$2.54±1.10^{Acd}$	$2.55±0.47^{Acd}$	$2.32±0.41^{Ad}$	$2.87±0.68^{Ac}$	$4.03±0.79^{Bb}$	$5.77±1.32^{ABa}$
	B	$2.71±0.44^{Ab}$	$2.75±0.49^{Ab}$	$2.59±0.46^{Ab}$	$3.19±0.70^{Ab}$	$4.94±0.76^{Aa}$	$5.31±1.19^{Ba}$
	M	$2.46±0.41^{Ac}$	$2.43±0.49^{Ac}$	$2.24±0.32^{Ac}$	$2.74±0.66^{Ac}$	$4.40±1.13^{ABb}$	$6.31±1.56^{Aa}$

^{A,B} different letters represent differences between groups ($P < 0.05$)
^{a,b,c} different letters represent differences between moments ($P < 0.05$)
P: primiparous (n = 26); B: biparous (n = 21); M: multiparous (n = 17)
