# Supplementary material for: Effect of Parity, Body Condition Score at Calving, and Milk Yield on the Metabolic Profile of Gyr Cows in the Transition Period
Source: Animals (Basel). 2023 Aug 3;13(15):2509. doi: 10.3390/ani13152509 (PMC10417048; doi:10.3390/ani13152509)
Supplement: Supplementary file 1 [file animals-13-02509-s001.zip › SupplementaryTable S2 Breda et al. Metabolic profile of Gyr cows Animals abr 2023.pdf]

Supplementary Table S2. Variation (mean ± SD) of serum concentrations of total proteins (TP) and albumin, and serum aspartate aminotransferase (AST) and gamma-glutamyltransferase (GGT) activities in high-producing Gyr cows grouped according to parity, on different days relative to calving (0 d), throughout the transition period.

Variable	Group	-21 d	-7 d	0 d	7 d	21 d	42 d
TP (g/L)	P	72.00±2.00^{Cb}	71.46±4.85^{Bb}	71.34±7.12^{Bb}	75.53±5.94^{Ba}	78.26±6.17^{Ba}	76.80±5.72^{Ba}
	B	77.70±5.19^{Bc}	76.40±6.32^{Ac}	77.30±5.95^{Ac}	78.05±5.73^{ABc}	82.55±4.00^{Ba}	82.45±3.70^{Aab}
	M	83.11±9.81^{Ab}	81.52±9.61^{Ab}	80.41±7.89^{Ab}	81.41±9.00^{Ab}	90.29±9.06^{Aa}	87.58±10.28^{Aa}
Albumin (g/L)	P	32.50±2.88^{Aa}	32.50±3.27^{Aa}	32.61±3.62^{Aa}	32.50±4.35^{Ba}	34.38±3.69^{Ba}	34.92±5.90^{Aa}
	B	32.75±2.33^{Ab}	34.40±2.47^{Aab}	34.90±2.46^{Aab}	35.40±6.18^{Aab}	37.35±4.82^{Aa}	37.05±4.47^{Aa}
	M	32.52±3.57^{Ab}	32.70±3.11^{Ab}	33.64±3.40^{Ab}	32.82±2.96^{ABb}	37.52±6.65^{Aa}	34.76±2.63^{Aab}
AST (U/L)	P	74.92±24.97^{Ab}	82.61±48.03^{Ab}	78.65±19.96^{Ab}	103.57±47.40^{Aa}	77.30±19.58^{Ab}	67.80±12.90^{Ab}
	B	61.40±11.02^{Ab}	64.85±15.93^{Bb}	72.15±11.78^{Ab}	93.20±27.67^{ABa}	71.35±12.70^{Ab}	72.40±14.31^{Ab}
	M	59.88±10.88^{Ab}	62.35±7.66^{Bb}	69.76±27.68^{Aab}	85.23±25.98^{Ba}	70.52±20.41^{Aab}	69.64±14.46^{Aab}
GGT (U/L)	P	27.50±3.78^{Ab}	32.11±4.36^{Ab}	49.53±30.92^{Aa}	33.53±9.19^{Ab}	30.26±6.35^{Ab}	26.19±4.82^{Ab}
	B	25.30±5.66^{Ab}	31.10±5.27^{Aab}	37.65±8.62^{Ba}	29.00±5.80^{Aab}	29.50±5.80^{Aab}	31.20±8.87^{Aab}
	M	27.88±6.31^{Ab}	32.00±6.43^{Aab}	42.11±15.75^{ABa}	37.23±27.40^{Aab}	38.41±25.49^{Aab}	33.47±9.34^{Aab}

^{A,B} different letters represent differences between groups ($P < 0.05$)
^{a,b,c} different letters represent differences between moments ($P < 0.05$)
P: primiparous (n = 26); B: biparous (n = 21); M: multiparous (n = 17)
