# Supplementary material for: Effect of Parity, Body Condition Score at Calving, and Milk Yield on the Metabolic Profile of Gyr Cows in the Transition Period
Source: Animals (Basel). 2023 Aug 3;13(15):2509. doi: 10.3390/ani13152509 (PMC10417048; doi:10.3390/ani13152509)
Supplement: Supplementary file 1 [file animals-13-02509-s001.zip › SupplementaryTable S3 Breda et al. Metabolic profile of Gyr cows Animals abr 2023.pdf]

Supplementary Table S3. Variation (mean ± SD) of serum concentrations of total calcium (Ca), phosphorus (P), and magnesium (Mg) in high-producing Gyr cows grouped according to parity, on different days relative to calving (0 d), throughout the transition period.

Variable	Group	-21 d	-7 d	0 d	7 d	21 d	42 d
Ca (mmol/L)	P	2.20±0.08^{Aab}	2.25±0.06^{Aab}	2.22±0.18^{Aab}	2.25±0.17^{Aab}	2.27±0.12^{ABa}	2.17±0.17^{Bb}
	B	2.46±0.23^{Ab}	2.29±0.24^{Aab}	2.20±0.49^{ABb}	2.37±0.45^{Aab}	2.50±0.37^{Aa}	2.27±0.28^{Aab}
	M	2.16±0.11^{Aab}	2.23±0.09^{Aab}	2.11±0.15^{Bb}	2.20±0.13^{Aab}	2.22±0.11^{Bab}	2.24±0.16^{ABa}
P (mmol/L)	P	2.46±0.23^{Aa}	2.29±0.24^{Aa}	1.81±0.49^{Ab}	2.37±0.45^{Aa}	2.50±0.37^{Aa}	2.27±0.28^{Aa}
	B	2.26±0.25^{Aa}	2.23±0.19^{Aa}	1.86±0.50^{Ab}	1.98±0.27^{Bab}	2.19±0.22^{Ba}	2.15±0.22^{Aa}
	M	2.24±0.31^{Aa}	2.35±0.30^{Aa}	1.67±0.52^{Ab}	2.07±0.46^{Ba}	2.13±0.29^{Ba}	2.16±0.31^{Aa}
Mg (mmol/L)	P	1.24±0.16^{Aa}	1.07±0.14^{Aa}	1.00±0.13^{Aa}	1.00±0.14^{Aa}	1.05±0.18^{Aa}	1.04±0.28^{Aa}
	B	1.12±0.09^{Aa}	1.04±0.12^{Aab}	0.91±0.10^{Ab}	0.97±0.10^{Ab}	1.08±0.10^{Aab}	1.06±0.08^{Aab}
	M	1.04±0.09^{Aa}	1.00±0.09^{Aab}	0.97±0.13^{Aab}	0.91±0.10^{Ab}	1.01±0.09^{Aab}	0.98±0.12^{Aab}

^{A,B} different letters represent differences between groups ($P < 0.05$)

^{a,b,c} different letters represent differences between moments ($P < 0.05$)

P: primiparous (n = 26); B: biparous (n = 21); M: multiparous (n = 17)
