# Supplementary material for: Effect of Parity, Body Condition Score at Calving, and Milk Yield on the Metabolic Profile of Gyr Cows in the Transition Period
Source: Animals (Basel). 2023 Aug 3;13(15):2509. doi: 10.3390/ani13152509 (PMC10417048; doi:10.3390/ani13152509)
Supplement: Supplementary file 1 [file animals-13-02509-s001.zip › SupplementaryTable S4 Breda et al. Metabolic profile of Gyr cows Animals abr 2023.pdf]

Supplementary Table S4. Variation (mean ± SD) of body condition score (BCS), serum concentrations of non-esterified fatty acids (NEFA), beta-hydroxybutyrate (BHB), and cholesterol, and plasma glucose concentrations in high-producing Gyr cows grouped according to BCS at calving, on different days relative to calving (0 d), throughout the transition period.

Variable	Group	-21 d	-7 d	0 d	7 d	21 d	42 d
BCS	HBCS	4.10±0.30^{Aa}	4.04±0.24^{Aa}	4.04±0.24^{Aa}	3.71±0.28^{Ab}	3.51±0.21^{Ac}	3.38±0.35^{Ac}
	NBCS	3.35±0.12^{Ba}	3.35±0.16^{Ba}	3.35±0.16^{Ba}	3.31±0.15^{Ba}	3.22±0.19^{Ba}	3.26±0.20^{Aa}
NEFA (mmol/L)	HBCS	0.28±0.18^{Ab}	0.49±0.43^{Ab}	0.90±0.36^{Aa}	0.84±0.61^{Aab}	0.54±0.32^{Ab}	1.09±0.73^{Aa}
	NBCS	0.36±0.17^{Ac}	0.49±0.44^{Abc}	0.91±0.30^{Aa}	0.77±0.47^{Aab}	0.50±0.33^{Abc}	0.69±0.49^{Babc}
BHB (mmol/L)	HBCS	0.34±0.12^{Ab}	0.44±0.10^{Ab}	0.47±0.14^{Ab}	0.69±0.25^{Aab}	0.55±0.22^{Ab}	0.86±0.57^{Aa}
	NBCS	0.36±0.13^{Ab}	0.44±0.14^{Ab}	0.50±0.10^{Aab}	0.68±0.24^{Aa}	0.46±0.20^{Aab}	0.66±0.52^{Bab}
Glucose (mmol/L)	HBCS	3.09±0.45^{Ab}	2.96±0.53^{Ab}	6.74±3.46^{Aa}	3.24±0.65^{Ab}	3.38±0.79^{Ab}	3.09±0.78^{Ab}
	NBCS	3.08±0.47^{Ab}	3.04±0.49^{Ab}	6.30±3.38^{Aa}	3.52±0.71^{Ab}	3.54±0.85^{Ab}	3.12±0.85^{Ab}
Cholesterol (mmol/L)	HBCS	2.67±0.44^{Ac}	2.61±0.51^{Ac}	2.46±0.42^{Ac}	2.94±0.73^{Ac}	4.77±1.15^{Ab}	6.69±1.43^{Aa}
	NBCS	2.55±0.47^{Ac}	2.54±0.55^{Ac}	2.34±0.42^{Ac}	2.86±0.42^{Ac}	4.65±1.20^{Ab}	6.38±1.49^{Aa}

^{A,B} different letters represent differences between groups ($P < 0.05$).

^{a,b,c} different letters represent differences between moments ($P < 0.05$).

HBCS: high body condition score (>3.5; n = 21); NBCS: normal body condition score (3.0-3.5; n = 20)
