# Supplementary material for: Effect of Parity, Body Condition Score at Calving, and Milk Yield on the Metabolic Profile of Gyr Cows in the Transition Period
Source: Animals (Basel). 2023 Aug 3;13(15):2509. doi: 10.3390/ani13152509 (PMC10417048; doi:10.3390/ani13152509)
Supplement: Supplementary file 1 [file animals-13-02509-s001.zip › SupplementaryTable S5 Breda et al. Metabolic profile of Gyr cows Animals abr 2023.pdf]

Supplementary Table S5. Variation (mean ± SD) of serum concentrations of total proteins (PT) and albumin, and serum aspartate aminotransferase (AST) and gamma-glutamyltransferase (GGT) activities in high-producing Gyr cows grouped according to BCS at calving, on different days relative to calving (0 d) throughout the transition period.

Variable	Group	-21 d	-7 d	0 d	7 d	21 d	42 d
TP (g/L)	HBCS	79.61±7.67^{Ab}	78.47±7.22^{Ab}	77.47±4.95^{Ab}	79.23±4.67^{Ab}	86.19±6.97^{Aa}	81.66±11.24^{Aab}
	NBCS	82.10±9.53^{Ab}	80.10±9.75^{Ab}	79.10±8.18^{Ab}	80.40±8.62$_{Ab}$	88.60±9.30^{Aa}	86.50±9.83^{Aa}
Albumin (g/L)	HBCS	32.28±2.72^{Ab}	33.33±2.70^{Ab}	34.85±2.63^{Aab}	35.00±6.06^{Aab}	37.04±6.41^{Aa}	35.47±5.81^{Aab}
	NBCS	32.55±3.30^{Ab}	32.95±2.96^{Ab}	33.90±3.29^{Aab}	33.30±2.97^{Ab}	37.30±6.13^{Aa}	35.00±2.53^{Aab}
AST (U/L)	HBCS	62.71±11.24^{Ab}	74.57±32.53^{Ab}	77.57±21.83^{Ab}	96.00±31.03^{Aa}	73.71±18.30^{Ab}	74.85±18.64^{Ab}
	NBCS	60.10±11.77^{Ab}	61.40±7.74^{Ab}	69.65±25.51^{Aab}	83.50±24.34^{Aa}	68.20±19.63^{Aab}	68.50±14.58^{Aab}
GGT (U/L)	HBCS	26.23±5.10^{Ac}	33.38±5.21^{Abc}	42.76±22.06^{Aa}	34.95±24.60^{Abc}	36.71±23.00^{Abc}	40.38±16.81^{Ab}
	NBCS	26.75±6.49^{Ab}	31.60±6.21^{Aab}	41.10±14.81^{Aa}	35.50±25.52^{Aab}	36.60±23.81^{Aab}	32.65±8.93^{Aab}

^{A,B} different letters represent differences between groups ($P < 0.05$).

^{a,b,c} different letters represent differences between moments ($P < 0.05$).

HBCS: high body condition score (>3.5; n = 21); NBCS: normal body condition score (3.0-3.5; n = 20)
