# Supplementary material for: Effect of Parity, Body Condition Score at Calving, and Milk Yield on the Metabolic Profile of Gyr Cows in the Transition Period
Source: Animals (Basel). 2023 Aug 3;13(15):2509. doi: 10.3390/ani13152509 (PMC10417048; doi:10.3390/ani13152509)
Supplement: Supplementary file 1 [file animals-13-02509-s001.zip › SupplementaryTable S6 Breda et al. Metabolic profile of Gyr cows Animals abr 2023.pdf]

Supplementary Table S6. Variation (mean ± SD) of serum concentrations of total calcium (Ca), phosphorus (P), and magnesium (Mg) in high-producing Gyr cows grouped according to BCS at calving, on different days relative to calving (0 d) throughout the transition period.

Variable	Group	-21 d	-7 d	0 d	7 d	21 d	42 d
Ca (mmol/L)	HBCS	2.23 ± 0.11^{Aa}	2.25 ± 0.10^{Aa}	2.17 ± 0.14^{Aa}	2.23 ± 0.13^{Aa}	2.27 ± 0.11^{Aa}	2.25 ± 0.19^{Aa}
	NBCS	2.18 ± 0.13^{Aab}	2.23 ± 0.08^{Aab}	2.12 ± 0.14^{Ab}	2.21 ± 0.14^{Aab}	2.26 ± 0.13^{Aa}	2.27 ± 0.17^{Aa}
P (mmol/L)	HBCS	2.24 ± 0.24^{Aa}	2.29 ± 0.30^{Aa}	1.74 ± 0.49^{Ab}	2.04 ± 0.38^{Aab}	2.16 ± 0.28^{Aa}	2.08 ± 0.24^{Aa}
	NBCS	2.23 ± 0.30^{Aa}	2.32 ± 0.28^{Aa}	1.73 ± 0.53^{Ab}	2.02 ± 0.44^{Aab}	2.17 ± 0.29^{Aa}	2.18 ± 0.30^{Aa}
Mg (mmol/L)	HBCS	1.07 ± 0.11^{Aa}	1.01 ± 0.15^{Aab}	0.98 ± 0.14^{Ab}	0.95 ± 0.11^{Ab}	1.03 ± 0.11^{Aab}	1.02 ± 0.12^{Aab}
	NBCS	1.06 ± 0.10^{Aa}	1.01 ± 0.09^{Aab}	0.96 ± 0.12^{Ab}	0.93 ± 0.10^{Ab}	1.02 ± 0.09^{Aab}	1.00 ± 0.12^{Aab}

^{A,B} different letters represent differences between groups ($P < 0.05$).

^{a,b,c} different letters represent differences between moments ($P < 0.05$).

HBCS: high body condition score (>3.5; n = 21); NBCS: normal body condition score (3.0-3.5; n = 20)
