# Supplementary material for: Effect of Parity, Body Condition Score at Calving, and Milk Yield on the Metabolic Profile of Gyr Cows in the Transition Period
Source: Animals (Basel). 2023 Aug 3;13(15):2509. doi: 10.3390/ani13152509 (PMC10417048; doi:10.3390/ani13152509)
Supplement: Supplementary file 1 [file animals-13-02509-s001.zip › SupplementaryTable S7 Breda et al. Metabolic profile of Gyr cows Animals abr 2023.pdf]

Supplementary Table S7. Variation (mean ± SD) of body condition score (BCS) and serum concentrations of non-esterified fatty acids (NEFA), beta-hydroxybutyrate (BHB), and cholesterol, and plasma glucose concentrations in Gyr cows grouped according to milk yield on different days relative to calving (0 d) throughout the transition period.

Variable	Group	-21 d	-7 d	0 d	7 d	21 d	42 d
BCS	HP	3.67 ± 0.43^{Aa}	3.64 ± 0.35^{Aa}	3.64 ± 0.35^{Aa}	3.51 ± 0.30^{Aa}	3.28 ± 0.28^{Ab}	3.29 ± 0.25^{Ab}
	MP	3.63 ± 0.43^{Aa}	3.64 ± 0.41^{Aa}	3.59 ± 0.51^{Aa}	3.45 ± 0.28^{Ab}	3.37 ± 0.25^{Abc}	3.26 ± 0.31^{Ac}
NEFA (mmol/L)	HP	0.28 ± 0.17^{Ac}	0.36 ± 0.28^{Abc}	0.92 ± 0.32^{Aab}	0.98 ± 0.64^{Aab}	0.65 ± 0.36^{Ab}	1.03 ± 0.64^{Aa}
	MP	0.31 ± 0.18^{Ac}	0.50 ± 0.47^{Ac}	0.87 ± 0.34^{Aa}	0.81 ± 0.56^{Aab}	0.52 ± 0.33^{Abc}	0.75 ± 0.56^{Bab}
BHB (mmol/L)	HP	0.39 ± 0.11^{Ac}	0.39 ± 0.09^{Ac}	0.49 ± 0.14^{Abc}	0.77 ± 0.23^{Aab}	0.63 ± 0.23^{Ab}	0.94 ± 0.66^{Aa}
	MP	0.35 ± 0.11^{Ab}	0.48 ± 0.12^{Aab}	0.46 ± 0.14^{Aab}	0.64 ± 0.25^{Aa}	0.50 ± 0.31^{Aab}	0.64 ± 0.36^{Ba}
Glucose (mmol/L)	HP	3.12 ± 0.55^{Ab}	2.95 ± 0.64^{Ab}	7.05 ± 3.50^{Aa}	3.02 ± 0.43^{Ab}	3.11 ± 0.74^{Ab}	2.77 ± 0.59^{Ab}
	MP	3.00 ± 0.54^{Ab}	2.98 ± 0.48^{Ab}	6.09 ± 2.89^{Ba}	3.58 ± 0.80^{Ab}	3.67 ± 0.79^{Ab}	3.42 ± 0.89^{Ab}
Cholesterol (mmol/L)	HP	2.73 ± 0.48^{Ad}	2.60 ± 0.42^{Ad}	2.54 ± 0.40^{Ad}	3.24 ± 0.56^{Ac}	5.16 ± 0.98^{Ab}	6.76 ± 1.54^{Aa}
	MP	2.55 ± 0.40^{Acd}	2.57 ± 0.56^{Ad}	2.40 ± 0.40^{Ad}	2.84 ± 0.86^{Ac}	4.61 ± 0.90^{Bb}	5.80 ± 1.24^{Ba}

[A,B] different letters represent differences betweeun groups ($P < 0.05$).

[a,b,c] different letters represent differences between moments ($P < 0.05$).

HP: high production (>30 kg/day; n = 22); MP: moderate production (20-30 kg/day; n = 30)
