# Supplementary material for: Effect of Parity, Body Condition Score at Calving, and Milk Yield on the Metabolic Profile of Gyr Cows in the Transition Period
Source: Animals (Basel). 2023 Aug 3;13(15):2509. doi: 10.3390/ani13152509 (PMC10417048; doi:10.3390/ani13152509)
Supplement: Supplementary file 1 [file animals-13-02509-s001.zip › SupplementaryTable S8 Breda et al. Metabolic profile of Gyr cows Animals abr 2023.pdf]

Supplementary Table S8. Variation (mean ± SD) of serum concentrations of total proteins (PT) and albumin, and serum aspartate aminotransferase (AST) and gamma-glutamyltransferase (GGT) activities in Gyr cows grouped according to milk yield on different days relative to calving (0 d) throughout the transition period.

Variable	Group	-21 d	-7 d	0 d	7 d	21 d	42 d
TP (g/L)	HP	81.36±10.08^{Abc}	79.81±8.46^{Ac}	78.86±7.35^{Ac}	80.09±7.29^{Ac}	85.59±8.33^{Aab}	86.77±10.63^{Aa}
	MP	79.56±5.89^{Ab}	78.73±7.49^{Ab}	77.86±5.87^{Ab}	78.53±6.21^{Ab}	85.66±6.28^{Aa}	81.50±6.53^{Bb}
Albumin (g/L)	HP	31.63±2.87^{Ad}	33.04±2.60^{Acd}	34.54±3.08^{Abcd}	35.22±5.88^{Abc}	39.13±8.01^{Aa}	37.18±5.84^{Aab}
	MP	33.36±2.67^{Ab}	33.90±2.97^{Ab}	34.90±2.39^{Aab}	34.76±5.30^{Aab}	36.73±3.24^{Ba}	35.33±3.30^{Aab}
AST (U/L)	HP	60.36±10.22^{Ab}	66.00±19.96^{Ab}	65.68±15.27^{Ab}	93.09±27.50^{Aa}	68.31±9.10^{Ab}	70.00±11.35^{Ab}
	MP	61.90±11.40^{Ac}	64.66±13.78^{Abc}	74.06±18.^{30Ab}	89.83±28.87^{Aa}	73.20±18.72^{Ab}	73.36±17.57^{Ab}
GGT (U/L)	HP	27.22±5.75^{Ac}	34.04±6.69^{Aabc}	41.81±19.45^{Aa}	30.31±5.47^{Abc}	32.90±5.60^{Aabc}	38.59±15.93^{Aab}
	MP	25.83±6.06^{Ab}	30.50±4.70^{Ab}	38.26±12.47^{Aa}	32.03±20.96^{Aab}	32.63±19.66^{Aab}	33.36±10.78^{Aab}

^{A,B} different letters represent differences betweeun groups ($P < 0.05$).
^{a,b,c} different letters represent differences between moments ($P < 0.05$).
HP: high production (>30 kg/day; n = 22); MP: moderate production (20-30 kg/day; n = 30)
