# Supplementary material for: Effect of Parity, Body Condition Score at Calving, and Milk Yield on the Metabolic Profile of Gyr Cows in the Transition Period
Source: Animals (Basel). 2023 Aug 3;13(15):2509. doi: 10.3390/ani13152509 (PMC10417048; doi:10.3390/ani13152509)
Supplement: Supplementary file 1 [file animals-13-02509-s001.zip › SupplementaryTable S9 Breda et al. Metabolic profile of Gyr cows Animals abr 2023.pdf]

Supplementary Table S9. Variation (mean ± SD) of serum concentrations of total calcium (Ca), phosphorus (P), and magnesium (Mg) in Gyr cows grouped according to milk yield on different days relative to calving (0 d) throughout of the transition period.

Variable	Group	-21 d	-7 d	0 d	7 d	21 d	42 d
Ca (mmol/L)	HP	2.23±0.09^{Aab}	2.29±0.09^{Aa}	2.15±0.14^{Ab}	2.22±0.15^{Aab}	2.30±0.10^{Aa}	2.31±0.11^{Aa}
	MP	2.19±0.12^{Aab}	2.22±0.09^{Aab}	2.17±0.13^{Ab}	2.23±0.12^{Aab}	2.27±0.13^{Aa}	2.26±0.16^{Aa}
P (mmol/L)	HP	2.32±0.24^{Aa}	2.33±0.20^{Aa}	1.55±0.44^{Bd}	1.87±0.28^{Bc}	2.04±0.24^{Bbc}	2.15±0.21^{Aab}
	MP	2.21±0.27^{Aa}	2.26±0.32^{Aa}	1.97±0.50^{Ab}	2.08±0.39^{Aab}	2.23±0.24^{Aa}	2.15±0.21^{Aab}
Mg (mmol/L)	HP	1.09±0.08^{Aa}	1.05±0.11^{Aab}	1.01±0.12^{Ab}	0.98±0.09^{Ab}	1.06±0.08^{Aab}	1.06±0.010^{Aab}
	MP	1.08±0.12^{Aa}	1.01±0.12^{Ab}	0.91±0.11^{Bc}	0.93±0.11^{Ac}	1.04±0.09^{Aab}	1.02±0.10^{Aab}

^{A,B} different letters represent differences betweeun groups (P < 0.05).

^{a,b,c} different letters represent differences between moments (P < 0.05).

HP: high production (>30 kg/day; n = 22); MP: moderate production (20-30 kg/day; n = 30)
